# Assessing the Usability of Web-Based Alcohol Education for Older Adults: A Feasibility Study

**DOI:** 10.2196/resprot.4545

**Published:** 2016-02-01

**Authors:** Arlene Fink, Lorna Kwan, Dan Osterweil, Jenna Van Draanen, Alexis Cooke, John C Beck

**Affiliations:** ^1^ Arlene Fink Associates Pacific Palisades, CA United States; ^2^ David Geffen School of Medicine Division of Geriatrics and Gerontology University of California, Los Angeles Los Angeles, CA United States; ^3^ David Geffen School of Medicine University of California, Los Angeles Los Angeles, CA United States

**Keywords:** alcohol, older adults, elderly, web-based, education, online alcohol education

## Abstract

**Background:**

Older adults can experience unfavorable health effects from drinking at relatively low consumption levels because of age-related physiological changes and alcohol’s potentially adverse interactions with declining health, increased medication-use and diminishing functional status. At the same time, alcohol use in older adults may be protective against heart disease, stroke, and other disorders associated with aging. We developed “A Toast to Health in Later Life! Wise Drinking as We Age,” a web-based educational intervention to teach older adults to balance drinking risks and benefits.

**Objective:**

To examine the intervention’s feasibility in a sample of community-dwelling current drinkers ≥55 years of age and examine its effects on their quantity and frequency of alcohol use, adherence to standard drinking guidelines, and alcohol-related risks.

**Methods:**

Participants were recruited in person, by mail and by telephone between September and October 2014 from a community-based social services organization serving Los Angeles County. Once enrolled, participants were randomly assigned to the intervention or to a control group. The conceptual frameworks for the intervention were the Health Belief Model, models of adult learning, and the US Department of Health and Human Services guidelines for designing easy-to-use websites. The intervention’s content focuses on the relationship between drinking and its effects on older adults’ medical conditions, use of medications, and ability to perform daily activities. It also addresses quantity and frequency of alcohol use, drinking and driving and binge drinking. The control group did not receive any special intervention. Data on alcohol use and risks for both groups came from the online version of the Alcohol-Related Problems Survey and were collected at baseline and four weeks later. Data on usability were collected online from the intervention group immediately after it completed its review of the website.

**Results:**

The 49 intervention and 47 control participants did not differ at baseline in age, ethnicity, medication use, medical conditions, or alcohol use and both groups were mostly female, college-educated, and in good health. Of the intervention participants, 94% (46/49) had little or no difficulty using the website, with 67% (33/49) reporting that they will change the way they think about drinking because of their exposure to the education. At the 4-week follow-up, the intervention group reported drinking less (*P*=.02). No changes between groups were found in quantity and frequency, adherence to recommended guidelines, or risk status.

**Conclusions:**

Community-dwelling older adults are receptive to online alcohol education. To be most effective, the education should be included as a component of a larger effort consisting of screening and counseling preferably in a health care setting.

## Introduction

Over 40% of US adults 65 to 74 years of age and 30% of adults 75 years and older are current drinkers [1,2]. Although the quantity and frequency of alcohol use tends to decline with age [1,3] consumption appears to be declining more slowly than in previous generations [4]. Older adults can experience unfavorable health effects even at relatively low consumption levels because of age-related physiological changes [5,6] and alcohol’s potentially adverse interactions with chronic illness, increased medication-use and diminishing functional status [2-3,6,7].

Alcohol is implicated in many medical problems common in older adults including hypertension [8-11], depression [12,13], breast cancer [14-16], and fractures [17,18]. More than 60% of older adults regularly use medications [19], many of which, such as nonsteroidal anti-inflammatory drugs (NSAIDs), anticoagulants, and sedatives, have the potential to interact adversely with alcohol [7,20-22]. In addition, baby-boomers may have patterns of substance use that differ from previous cohorts resulting in a greater likelihood of combined alcohol and recreational drug use leading to increased risk for emergency department use and hospitalization [23-25]. Binge drinking (more than 4 or 5 drinks at one sitting), which greatly increases the chances of injury to self or others due to car crashes, violence, and suicide [26], is a serious problem in older adults who drink less intensely per occasion than their younger counterparts but binge more frequently than any other age group [26].

About 14.5% of older adults drink in excess of the National Institute on Alcohol Abuse and Alcoholism’s (NIAAA) recommended limits [27], placing them at risk for alcohol-related problems. When health and drinking patterns are taken into account, about half of all drinkers 65 years of age or older may be at risk for experiencing alcohol-related harm even if they drink within recommended limits [2]. Considering that the number of older adults will increase to more than 20% of the US population by 2030 [28], the number of older people with alcohol-related risks and problems will increase even if drinking prevalence remains constant.

Accurate evaluations of alcohol-related problems in older persons are somewhat complicated by evidence that moderate consumption may have beneficial effects on cardiovascular functioning, stroke-prevention and all-cause mortality [29-36]. When compared to abstention, light to moderate drinking, such as 1 to 6 drinks weekly, is associated with a lower risk of incident dementia [37], congestive heart failure [38], rheumatoid arthritis [39], and diabetes [40,41].

Research and education tend to focus on alcohol use disorders, especially among younger people, and older adults usually do not have direct access to pertinent instruction that emphasizes drinking’s potential benefits and risks that are specific to aging. Nor are data available on whether online alcohol education is usable by and acceptable to older adults, or if it can influence their drinking risks.

In response to the need for age-appropriate alcohol education, we developed and evaluated the feasibility of “A Toast to Health in Later Life! Wise Drinking as We Age,” a Web-based program to educate older adults about the risks and potential benefits of drinking associated with aging. The Internet has been recognized for many years as an important mechanism for supplementing direct medical care [42-45]. Older adults are active Web users, with nearly 80% of persons 55 to 64 years and 60% of those 65 and older going online at least once a day [46]. Over two-thirds of seniors in their 70s are Internet users, and more than half use broadband [47].

People use the Internet because of several favorable features: the convenience of being able to search quickly for information at any time, unlimited access to inexpensive information, self-pacing, and user anonymity when searching for subject-sensitive health information [48-52]. This study evaluated “A Toast to Health in Later Life’s” feasibility and influence on alcohol use and risks for alcohol-related problems in a sample of older current drinkers living independently in the community.

## Methods

### Participants and Setting

Participants were eligible for this study if they (1) were 55 years of age or older; (2) had 1 or more drinks containing alcohol in the past 3 months; (3) had an email account and were willing to share their email address so that staff could provide links to the educational program and study assessments; (4) were comfortable using the Internet; (5) had access to high-speed Internet; and (6) were willing to spend about 10 to 30 minutes on 2 separate occasions 4 weeks apart to complete an online alcohol education program and answer online questions in English about alcohol and health. Participants were recruited between September and October 2014 at a non-profit community-based social services organization located in Santa Monica, California and serving Los Angeles County. A trained study site coordinator used a combination of methods to recruit participants. These included in-person contacts with potential participants, mailings, and phone messages to the organization’s database of volunteers and users. The organization’s Chief Executive Officer also emailed qualified staff and colleagues with information about the study and requested their participation. Interested persons responded directly to the study’s site coordinator and the CEO was not told who responded.

### Study Design

The study site coordinator screened interested participants for their eligibility using a standardized script. Using a random number generator [53], we produced 200 numbers in random order. These numbers were allocated to each eligible study participant in the sequential order that they were generated. Participants with odd numbers were assigned to the intervention group, and those with even numbers were allocated to the control group. For example, the first 4 randomly generated numbers were 136, 172, 187, and 61. This meant that the first 2 eligible participants were assigned to the control group, whereas the next 2 participants were assigned to receive “A Toast to Health in Later Life!”

Participants in the intervention group received the URL for “A Toast to Health in Later Life!”, a study user name, and a password. Once in the site, intervention participants were given the choice of watching a video or reading a transcript that explained the study’s purposes and methods. If still interested, they clicked “submit” and were automatically directed to the informed consent form. After consenting, participants were automatically directed to the baseline survey, which collected information on drinking, health, medication use, functional status, lifestyle, and demographics. Participants were sent back to “A Toast to Health in Later Life!” automatically upon survey completion.

We programmed the website so that participants were required to review the entire site. Once they completed the review, however, participants were able to go back to any section that interested them. Intervention participants were given a brief post-survey focusing on the site’s usability.

Control group participants completed the same online informed consent and baseline survey as the intervention group. They were offered access to the site after the completion of data collection.

The study’s site coordinator emailed a link to the follow-up survey to all participants 4 weeks after each completed a baseline survey. Intervention and control participants completed identical follow-up surveys. Participants were reimbursed $35 after they completed all required data collection activities.

### Conceptual Frameworks

The conceptual frameworks for this study and for use in guiding website content, questionnaire development, data collection and data analysis were The Health Belief Model, models of adult learning, and the US Department of Health and Human Services (DHSS) guidelines for designing easy-to-use websites [54].

We selected The Health Belief Model because it provides a framework for specifying personal and situational factors likely to influence health behavior [55], and it is among the most rigorously studied and commonly used models to study how education changes behavior [55-59]. According to the model, changing behavior is contingent upon several key factors including knowledge of the problem and its consequences and self-efficacy to do something about the problem. Throughout the website, we included facts (“Did You Know?”) and practice exercises to reinforce learning. To strengthen self-efficacy, the site provides separate pages on how to cut down on drinking, how to speak to a doctor or other health professional about alcohol, and where to go for help. We used selected behavior change techniques, such as instruction on how to perform a behavior and demonstration of the behavior [60,61] to reinforce knowledge and self-efficacy.

To encourage learning, we relied on theories of adult learning which are often derived from or based on Knowles’ theory, which he called “andragogy” [62,63]. Knowles’ theory distinguishes adult from other learning in its emphasis on active learning, self-pacing, and problem-centered concepts [64,65]. The Internet is well suited to active learning and self-pacing. To facilitate active learning, “A Toast to Health in Later Life!” includes practice exercises and feedback.

We used DHSS guidelines [54] for easy-to-use websites (eg, keeping navigation simple and consistent; minimizing scrolling) to guide the website’s development and separated the graphics arts component of Web development from the technical one so as to ensure that the site’s graphics were colorful and age appropriate regardless of any technical challenge. The 2 study team geriatricians (JCB and DO), who have expertise in educating older learners, independently reviewed the website twice to make certain that it conformed to DHSS’ usability criteria.

### The Intervention

#### Content

“A Toast to Health in Later Life! Wise Drinking as We Age” is a Web-based education program that aims to teach older adults how to balance the benefits and risks of drinking. The website was developed by the study’s team of alcohol researchers (AF and JCB), experts in geriatric medicine and education (JCB and DO), a computer programming expert (GY), and a Web designer (MR).

The website’s intellectual content is derived from work done in connection with The Alcohol-Related Problems Survey (ARPS). The ARPS is a screening and education system that provides older adults and their physicians with tailored feedback on their alcohol-related risks based on their responses to a survey of alcohol use and health [66-73]. The ARPS system includes an educational component, which was developed with the assistance of over 200 community-dwelling older adults and then tested for usability in primary care practice with more than100 participants [74,75]. The study’s geriatricians (JCB and DO) adapted the ARPS’ educational content to “A Toast to Health in Later Life!” and used their expert knowledge and clinical experience to ensure the timeliness and relevance of the subject matter across the aging spectrum.

“A Toast to Health in Later Life!’” contains 9 sections, each of which provides information to achieve specific instructional objectives based on the ARPS’ framework and content. For example, one objective is “to describe the relationship between drinking and medical conditions, medication use, and functional status in older adults.” This objective is addressed throughout the site, but is emphasized in Section 1 of the website, “Thinking about Drinking and Aging.” Sample content includes a statement such as: “Alcohol affects the workings of common medicines like high blood pressure medicines, pain killers, and antihistamines. Alcohol can also worsen or cause health problems such as cancer, heart disease, and depression. Also, some older people have problems walking, sleeping, or remembering things. Alcohol complicates the care of these problems.”

**Figure 1:**
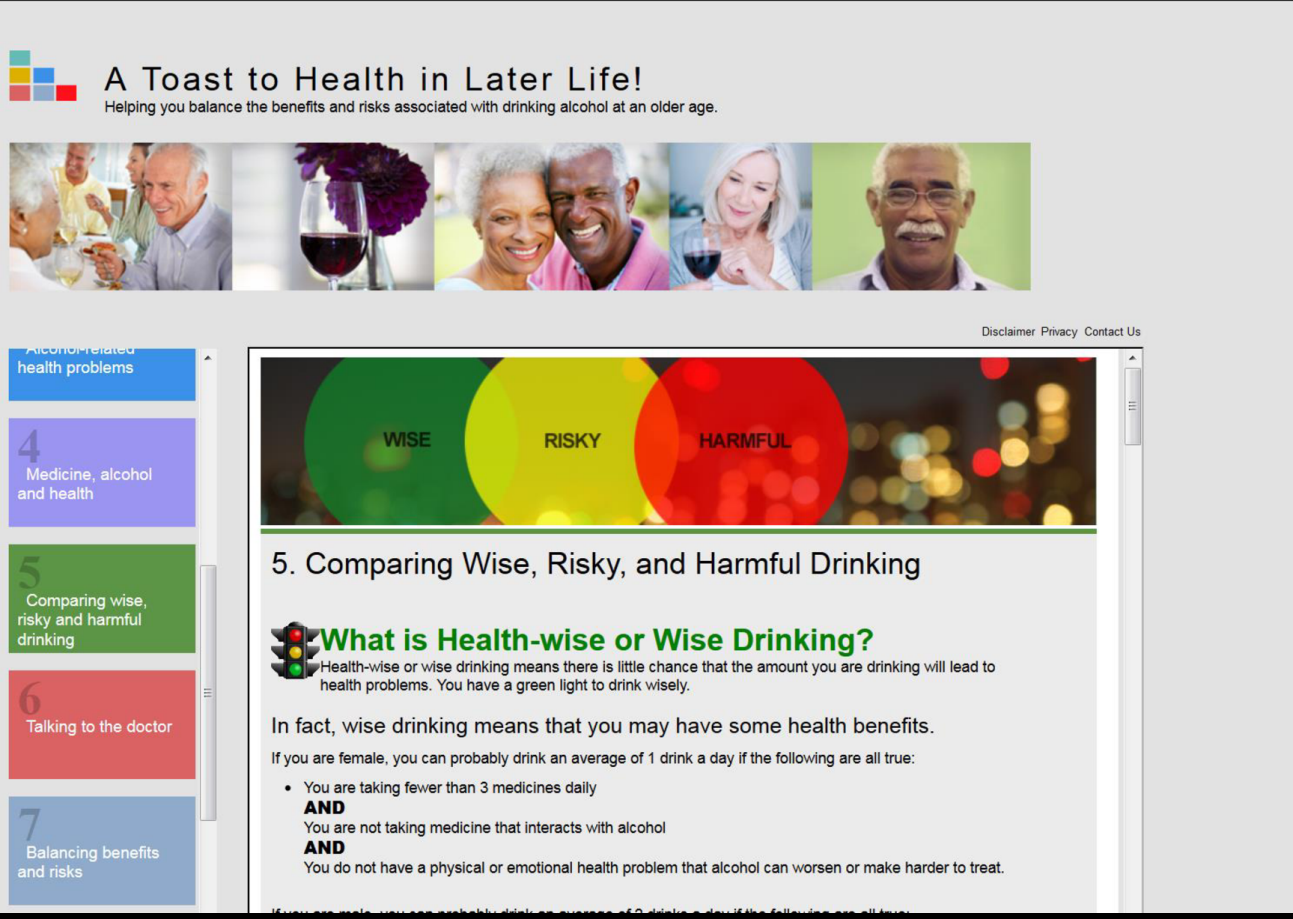
Screenshot from "A Toast to Health in Later Life! Wise Drinking as We Age". Content for one instructional objective.

Figure 1 contains a screenshot showing a portion of the content for the instructional objective, “to compare nonhazardous (wise), hazardous (risky) and harmful drinking.”

#### The Control

The control group did not receive any special intervention. Comparable patient education for older adults was not available, and we did not have the resources to develop an alternative and test it for feasibility and comparability.

#### Website Pilot Test

We conducted a 3-phase pilot test of the website among 21 people. The pilot test’s purpose was to produce a website that was appropriate in content and usability and ready for a more comprehensive feasibility test. Participants in all phases of the pilot test met the same eligibility criteria as participants in the main feasibility study. The study’s site yielded 17 participants, and 4 were recruited through personal contact. In the first phase of the pilot, we tested a prototype of the website on 5 older adults because evidence suggests that the best results come from testing no more than 5 users, and running as many small tests as you can [76]. The test asked participants to review the site without assistance. The study team (AF and JCB) then interviewed them in person or on the phone about the site’s usability, appropriateness, and potential benefit. We revised the site based on participants’ advice and retested it on 5 additional people who stated that they found the site easy to navigate, they learned from it, and they were confident that they could answer questions about alcohol use and aging. In the third phase of the pilot test, 4 people reviewed the website and answered questions about usability, while an additional 7 people reviewed the website and completed all study instruments.

#### Access to the Site

Access to the website required a user name (email address) and password (a-zA-z0-9) that was provided by the System Administrator. The System Administrator could grant System Administrator Privileges to others.

“A Toast to Health in Later Life!” was developed in Microsoft Razor MVC3 running under IIS.  It is a dynamic website that presents information to users based on their progress through the site and their security profile.  Beginning and end study surveys were hosted by Survey Monkey and embedded in the site's pages. The surveys were accessible to both the intervention and control groups, although the control group was not able to access any of the content pages.

### Outcomes and Measures

#### Demographics

We used standard questions to ask participants at baseline about their sex, race and ethnicity, birthdate, and education. Participants were asked to rate their health status as being excellent, very good, good, fair, or poor.

#### Alcohol Use and Risks

**Figure 2:**
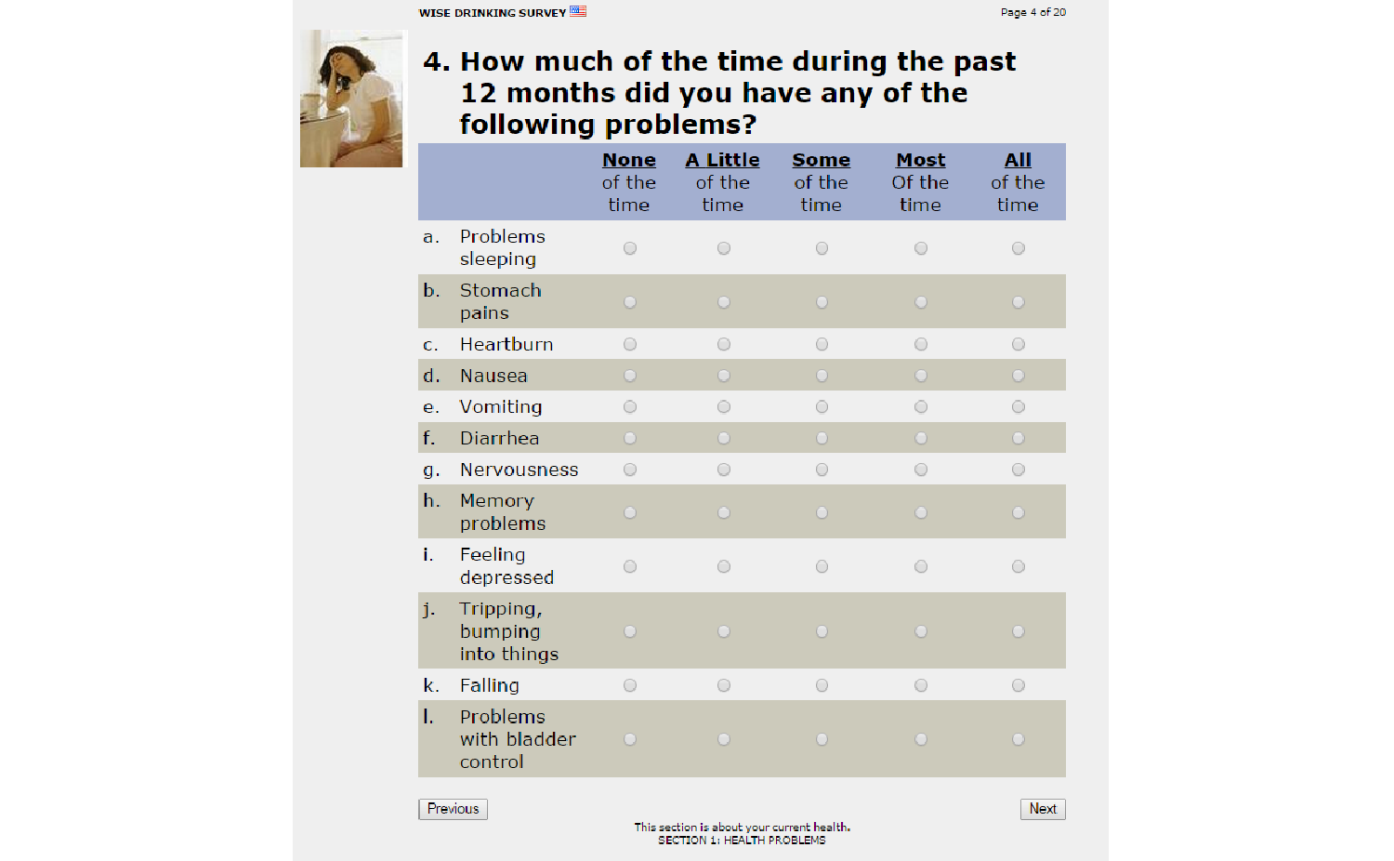
Sample question from the Alcohol-Related Problems Survey (ARPS).

To measure their alcohol-related use and risks, all participants completed the ARPS at baseline and 4 weeks after enrollment into the study. Since its development in the 1990’s, the ARPS and its derivatives have been used with thousands of older adults in community and research settings to study alcohol’s use and risks [2,67,77,78]. The ARPS’ development, psychometric properties, and research use are well-documented [2,67,68,70,71,79]. A sample question from the ARPS is given in Figure 2.

The ARPS uses terminology [80,81] to classify alcohol-related health risk into 3 categories: harmful (consumption that may exacerbate or complicate existing alcohol-related problems), hazardous (consumption that poses risks of future harm for individuals with specific medical conditions, functional status, or symptoms, taking specific medications, or engaging in risky behaviors such as smoking), and nonhazardous (neither harmful nor hazardous and potentially beneficial).

The ARPS’ scoring algorithms have been updated with the system’s continued use in the United States and in other countries [82,83]. The algorithms first consider a person’s reported quantity and frequency of consumption in relation to each of 63 factors (eg, medication use, binge drinking). The specific consumption patterns that confer risk vary substantially depending on the factor being considered. A sample of the tables and specifications for scoring the ARPS can be found in Multimedia Appendix 1.

To determine if NIAAA recommendations for older drinkers were exceeded [27], women and men 65 years of age and older who reported drinking 7 or more drinks weekly were considered at risk. All men under 65 years who drank 14 or more weekly drinks were also considered at risk.

#### Usability

The intervention participants were automatically directed to a usability survey immediately upon completing their website review. The survey asked if participants learned about alcohol and health; if they were confident they could now answer questions about alcohol and health; and if they were confident, whether the website contributed to their confidence. We also asked participants if the website changed the way they now think about drinking, if they had any difficulty using the website and if they would recommend it to others.

#### Ethics and Informed Consent

Eligible participants were asked to complete online consent after they were given the opportunity to listen to and read an overview of the study’s purposes and procedures. The informed consent took the form of an information sheet with the following categories: the study’s purposes and methods, a description of participant expectations, a discussion of possible risks or discomforts associated with participation, a discussion of possible benefits, payment for participation, measures used to protect privacy, whom to contact if questions arose, rights as a research subject, and participants’ right to quit the study at any time. Participants who clicked on submit after reading information sheet were automatically directed to the study’s baseline survey. Participants were free to close their browsers at any time, thus leaving the study, but intervention participants who completed the baseline survey and also agreed to continue, clicked on a link that automatically directed them to the study’s website. Entry to all study materials was protected by password and all responses were collected over secured, encrypted SSL/TLS connections. An independent Institutional Review Board (US Office of Human Subjects Research IRB #0000667) approved this study. A copy of the informed consent form can be found in Multimedia Appendix 2.

#### Data Analysis

Analyses of outcome data were based on the per-protocol method which is commonly used in feasibility studies because of their exploratory nature. Thus, data were analyzed for the 47 control and 49 intervention group participants who provided complete data on all outcome measures.

The outcomes were measured 4 weeks after enrollment and included (1) the quantity and frequency of drinking as assessed by the ARPS; (2) the percentage of participants drinking above NIAAA recommended levels; (3) the percentage of participants who were harmful, hazardous and nonhazardous drinkers as assessed by the ARPS; and (4) whether participants report changing their drinking amount in the past 4 weeks as indicated by their answers to survey questions.

Baseline characteristics are reported for the intervention and control groups. Categorical data are reported as frequencies (percentages), continuous data are reported as means and standard deviations (SD), and non-normally distributed data are reported as medians and ranges. We used Chi-square tests (or Fisher’s exact tests) for categorical data and *t* tests for continuous data (or Wilcoxon-Mann-Whitney tests for non-normally distributed data) to analyze differences between the 2 groups. To determine any changes from baseline to follow-up, we used Chi-square tests (or Fisher’s exact tests) to test group distributions at baseline versus follow-up. As a sensitivity analysis, we excluded participants who were nonhazardous at baseline to compare changes among hazardous and harmful drinkers. We also ran paired data analyses with McNemar’s test (or Bowker’s test of symmetry as appropriate) to determine any changes from baseline to follow-up within the intervention and control groups.

All tests were 2-sided, and statistical significance was set at .05. All analyses were conducted with SAS 9.4 (SAS Institute).

## Results

### Study Participants

**Figure 3:**
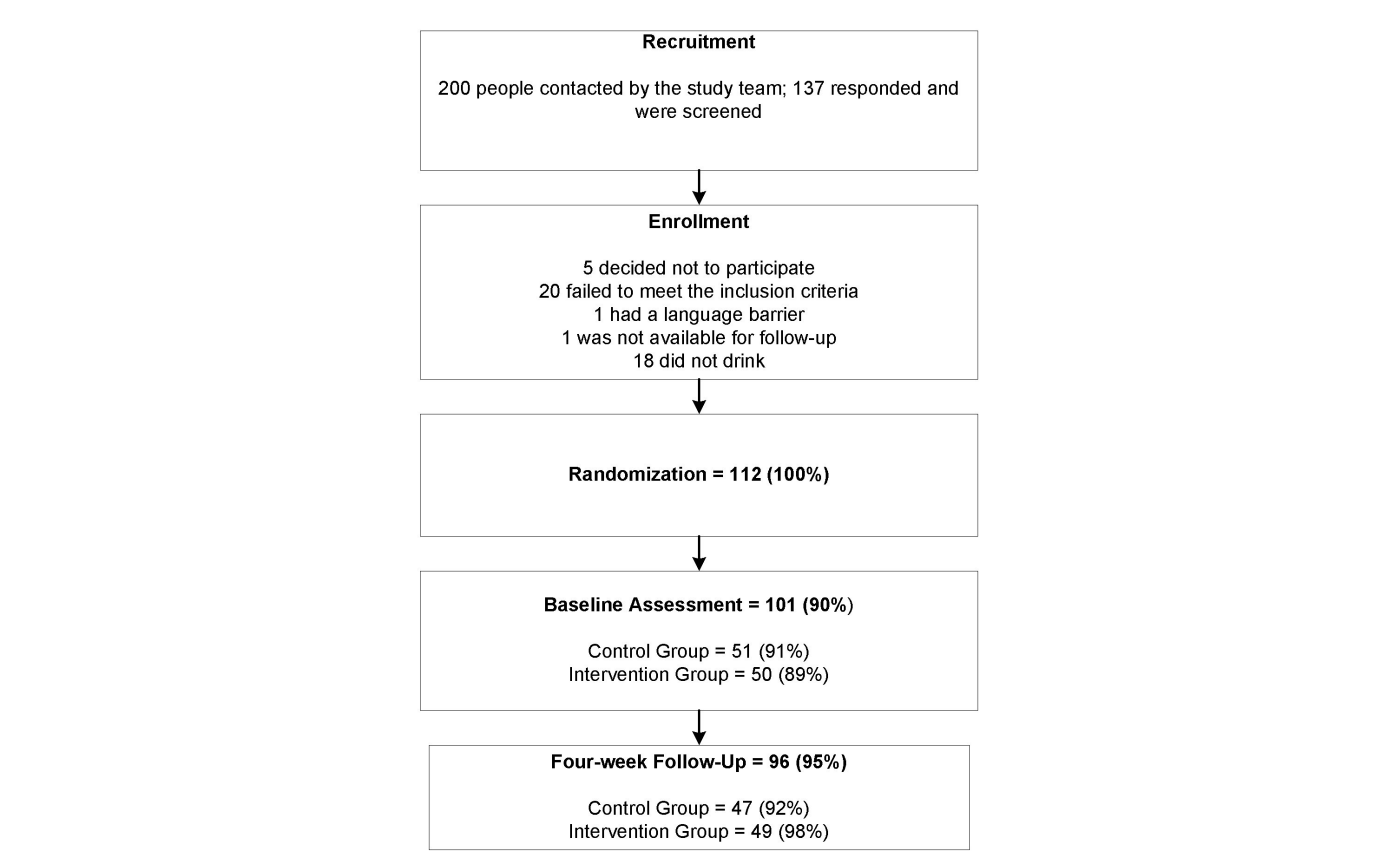
Participant flow chart.

Over the study’s 3-month recruitment period, we screened 137 interested participants (Figure 3), 20 of whom were not eligible for participation. There were no differences between control and intervention groups in enrollment or attrition. Of the 112 participants who were randomized, 91.0% (51/56) of the control and 89.0% (50/56) of the intervention group completed the baseline assessment. No differences were found in sources of recruitment for participants who completed the baseline assessment, with 72.5% (37/51) of the control and 72.0% (36/50) of the intervention having been recruited from the study site’s volunteers. Four weeks after enrollment, 92.0% (47/51) of the control and 98.0% (49/50) of the intervention group completed all study measures.

The intervention and control groups did not differ in gender; age; education; ethnicity; or alcohol use at baseline, and they were mostly female; college educated; and in good health.

**Table 1:** Intervention and control participant baseline characteristics.

	Intervention Group (n=49) n (%)	Control Group (n=47) n (%)	*P*
Sex (Female)	34 (69.4)	33 (70.2)	.93
Education, % College or more	39 (79.6)	41 (87.2)	.33
Health status, % Excellent or very good	37 (75.5)	31 (66.0)	.30
Race: White	44 (89.8)	42 (89.4)	.99^a^
Ethnicity: Hispanic	3 (6.1)	3 (6.4)	
Doctor ever told you have hypertension	21 (42.9)	21 (44.7)	.86
Doctor ever told you have breast cancer (♀)	4 (11.8)	5 (15.2)	.74^a^
Doctor ever told you have depression	6 (12.2)	8 (17.0)	.51
Falls were a problem in the past 12 months	6 (12.5)	8 (17.0)	.53
Daily NSAIDs^c^	13 (26.5)	12 (25.5)	.91^a^
Daily high blood pressure medicine	20 (40.8)	17 (36.2)	.64
Daily take an anticoagulant	1 (2.1)	1 (2.2)	.99^a^
Weekly sedative, narcotic or tranquillizer	11 (22.5)	8 (17.0)	.50
Ever drank 4 or more drinks at one sitting in past 12 months	8 (16.3)	11 (23.4)	.38

^a^ Fisher exact test

^b^ Wilcoxon-Mann-Whitney test

^c^ NSAID: Nonsteroidal anti-inflammatory drug

On average, participants in each group were 70 years of age and White. In the intervention group 42.9% (21/49) participants reported that their doctor told them they had hypertension and 40.8% (20/49) were taking antihypertensives. Similarly, 44.7% (21/47) of the controls reported having been told they had hypertension, and 36.2% (12/47) were using antihypertensives. Of the 13 intervention participants 26.5% (13/49), and 12 controls 25.5% (12/47) used NSAID’s daily. Weekly drinking varied widely, with members of the intervention group drinking from 0 to 28 drinks, and members of the control group drinking from 0 to 21 drinks. Of the intervention group, 16.3%) (8/49) of the intervention and 23.4% (11/47) of the control group participants drank 4 or more drinks at one sitting in the past 12 months.

### Drinking Quantity and Frequency

Drinking quantity did not change over time for the intervention (*P*=.31) or control group (*P*=.56) (Table 2).

At baseline, 89.8% (44/49) of the intervention and 97.8% (46/47) of the control group drank 2 drinks or less when they drank, and at follow-up, 96.0% (47/49) of the intervention and 93.6% (44/47) of the control group drank 2 drinks or less. Similarly, the frequency with which study participants drank did not change significantly from baseline to follow up for the intervention (*P*=.99) or the control group (*P*=.67). Paired data analyses yielded similar results for quantity and frequency of drinking (all *P*-values >.05).

### Adherence to NIAAA Recommended Weekly Limits

The proportion of participants who exceeded NIAAA’s recommended weekly limits did not significantly change over time in the intervention (*P*=.99) or control (*P*=.53) group (Table 3). At follow-up, 30.6% (15/49) of the intervention and 48.9% (23/47) of the control group’s drinking exceeded the NIAAA’s limits. No differences were found in the paired data analyses.

**Table 2:** Quantity and frequency of drinking.

Quantity/Frequency	Intervention	Control					
		Baseline (n=49) n (%)	Follow-up (n=49) n (%)	*P*	Baseline (n=47) n (%)	Follow-up (n=47) n (%)	*P*
**Quantity**							
	3 or more drinks per day^a^	5 (10.2)	2 (4.1%)	.31^b^	1 (2.1%)	3 (6.4%)	.56^bd^
	2 drinks per day	9 (18.4)	14 (28.6%)		16 (34.0%)	18 (38.3% )	
	1 drink or less	35 (71.4)	33 (67.4%)		30 (63.8%)	26 (55.3%)	
**Frequency**							
	Drinks daily or almost daily^c^	15 (30.6)	14 (28.6)	.99^b^	18 (38.3)	18 (38.3)	.67^d^
	Drinks 4 or 5 times a week	4 (8.2)	5 (10.2)		6 (12.8)	9 (19.2)	
	Drinks 2 or 3 times a week or less	30 (61.2)	30 (61.2)		23 (48.9)	20 (42.6)	

^a^ No differences in quantity between the groups at baseline (*P*=.09) or follow-up (*P*=.54).

^b^ Fisher’s exact test

^c^ No differences in frequency between groups at baseline (*P*=.46) or follow-up (*P*=.17).

^d^ *P*-values for the paired data analyses (Bowker's test of symmetry) between baseline and follow-up for quantity is .26 for the intervention group and .26 for the control group; for frequency, the *P*-value is .80 for the intervention group and .36 for the control group.

**Table 3:** Participants who passed and failed to meet NIAAA’s^a^ recommended weekly limits.^b^

Result	Intervention			Control		
	Baseline(n=49) n (%)	Follow-up(n=49) n (%)	*P* (by time)	Baseline (n=47) n (%)	Follow-up (n=47) n (%)	*P* (by time)
NIAAA fail^c^	15 (30.6)	15 (30.6)	.99^d^	20 (42.6)	23 (48.9)	.53^d^
NIAA pass	34 (69.4)	34 (69.4)		27 (57.4)	24 (51.1)	

^a^ NIAAA: The National Institute on Alcohol Abuse and Alcoholism

^b^ 7 drinks weekly for all women and men 65 years of age and older; 14 drinks weekly for men under 65 years of age

^c^ No differences between groups at baseline (*P*=.22) or follow-up (*P*=.67).

^d^ *P*-values for the paired sample analyses (McNemar’s test) between baseline and follow-up are .99 (100% agreement) for the intervention group and .26 for the control group.

### Change in Drinking Risk

At baseline, 44.9% (22/50) of intervention and 66.7% (31/51) of control group were harmful or hazardous drinkers (Table 4). No changes occurred from baseline to follow-up for either group in any category. There were also no differences in the risk categories between intervention and control at either baseline or follow-up (*P*=0.12 and *P*=0.11, respectively). This remained true in the sensitivity analysis where we excluded the participants that were nonhazardous drinkers at baseline (*P*=0.94 at baseline and *P*=0.58 at follow-up) (data not shown).

**Table 4:** Drinking risk.

Risk level	Intervention			Control		
	Baseline^a^ (n=49) n (%)	Follow-up^a^ (n=49) n (%)	*P*	Baseline^a^ (n=47) n (%)	Follow-up^a^ (n=47) n (%)	*P*
Harmful^c^	13 (26.5)	16 (32.7)	.63	18 (38.3)	21 (44.7)	.74^b^
Hazardous^d^	9 (18.4)	6 (12.2)		13 (27.7)	10 (21.3)	
Non-hazardous^e^	27 (55.1)	27 (55.1)		16 (34.0)	16 (34.0)	

^a^ No differences in risk category at baseline (*P*=.12) or follow-up (*P*=.11).

^b^ *P*-values for the paired data analyses (Bowker’s test of symmetry) between baseline and follow-up are .73 for the intervention and .72 for the control group.

^c^ Problems are likely

^d^ At-risk for problems

^e^ No known risks

### Change in Drinking From Baseline to Follow-up

Participants were asked if they changed their drinking in the past 4 weeks, and if so, whether they drank more, the same, or less. Table 5 shows that although intervention group did not differ from control group at baseline (*P*=.74), the intervention group differed significantly from the control group at follow-up (*P*=.02), with intervention participants reporting that they drank less.

**Table 5:** Participants reported change in drinking in the past 4 weeks.

	Baseline		*P*	Follow-up		*P*
	Baseline^a^ (n=49) n (%)	Follow-up^a^ (n=47) n (%)	.74	Baseline^a^ (n=49) n (%)	Follow-up^a^ (n=47) n (%)	.02^b^
Less	6 (12.2)	4 (8.5)		13 (26.3)	4 (8.5)	
Same	0 (0.0)	0 (0.0)		0 (0.0)	0 (0.0)	
More	43 (87.8)	36 (73.5)		37 (73.5)	43 (91.5)	

^a^The Fisher exact test *P* value for the control group from baseline to follow-up is .99.

^b^ *P* value for the paired data analyses (McNemar’s test or Bowker’s test of symmetry) is .02 for the intervention group from baseline to follow-up; for the control group, the *P*-value is .99.

### Usability

On average, the 49 intervention participants spent between 10 and 20 minutes on the website. The majority (93.9%, 46/49) reported having little or no difficulty using the site (Table 6). Most also reported (96.0%, 47/49) reported that they learned from the website, and 75.5% (37/49) stated being very or somewhat confident that they can answer questions about alcohol and health. A large proportion (77.6%, 38/49) indicated that the website helped them to become more confident in their ability to answer questions, and 93.9% (46/49) definitely or probably would recommend the site to others. Finally, 67.3% (33/49) said the use of the site will change the way they think about drinking.

**Table 6:** Intervention participants’ report on the usability of “A Toast to Health in Later Life!” (n=49).

Usability characteristic		n (%)
**Difficulty using the site**		
	Rated “No difficulty”	36 (73.5)
	Rated “Little difficulty”	10 (20.4)
**Learned from the site**		
	Rated “A great deal”	32 (65.3)
	Rated “A little”	15 (30.6)
**Confident can answer questions about alcohol**		
	Rated “Very”	12 (24.5)
	Rated “Somewhat”	25 (51.0)
**Website increased confidence**		
	Rated “Definitely”	18 (36.7)
	Rated “Probably”	20 (40.8)
**Recommend to others**		
	Rated “Definitely”	30 (61.2)
	Rated “Probably”	16 (32.7)
**Change the way you think about drinking**		
	Rated “Some”	32 (65.3)
	Rated “Much”	1 (2.0)

## Discussion

### Principal Results

We developed and tested the feasibility of a “Toast to Health in Later Life!” with older adults who were comfortable using the Internet and lived independently within the community. Most intervention participants had little or no difficulty navigating the website and recommended it to others. The participants reported that they learned from the site, could now confidently answer questions about alcohol and aging, and agreed that the information they gathered will change the way they think about drinking. Intervention participants reported drinking significantly less than controls 4 weeks after study enrollment.

Despite the intervention group’s favorable review of the website, and their perception that they drank less because of it, we found no difference between the intervention and control participants in their quantity and frequency of drinking, adherence to NIAAA weekly drinking guidelines, or drinking risks over the study’s 4-week data collection period.

There are several explanations for the discrepancy between participants’ favorable perceptions and the study’s statistical findings. First, notwithstanding the site’s appeal to participants, the study may not have provided the supportive resources necessary for them to change their drinking behavior. Other successful efforts to reduce hazardous and harmful drinking in older adults have provided access to screening, personalized feedback, health care provider advice and other services in addition to education. In Project GOAL [84], one of the first studies to target older adults, physicians used a work-book to give advice to patients, included a second reinforcement visit with the physician, and provided 2 follow-up calls from a clinic nurse 2 weeks after each physician visit. These features probably contributed to its success in reducing both 7-day alcohol use and binge drinking.

Using the ARPS screening and education system, a study of 665 older adults and 23 physicians in the same community as the present study provided screening and personalized feedback to patients and their physicians in addition to patient education. Patients significantly reduced harmful drinking at follow-up from a statistically expected 21% in usual care to 16% and increased nonhazardous drinking from 52% expected in usual care to 58% over a 12-month period [67].

Another study of 1186 community-dwelling older drinkers and 31 primary care providers [77] succeeded in reducing at-risk drinking as defined by a measure based on the ARPS. In addition to providing participants with educational materials, this study also provided participants with personalized reports, drinking diaries, physician advice during office visits, and telephone counseling delivered by a health educator. Similarly, a study [78] which included 631 older at-risk drinkers, also using measures derived from the ARPS algorithms, found that its multi-faceted intervention among older at-risk drinkers in primary care did not reduce the proportions of at-risk or heavy drinkers but the intervention was found to reduce the amount of drinking at 12 months [77]. This study’s participants were given personalized reports, a booklet on alcohol and aging, a drinking diary, and received advice from a primary care provider and telephone counseling from a health educator.

These studies took place in medical rather than community settings. The presence and support of health personnel is likely to have motivated participants, encouraging them to change their drinking behavior. The effective use of health personnel to motivate patients has been shown in clinical settings for problems such as preventive health care use, health behaviors, and control of diabetes and hyperlipidemia [85,86].

Another possible explanation of the present study’s inability to detect differences in quantity and frequency and in risks is that its brief duration may have been insufficient for change to occur. Many participants may not have seen a health care provider over the study’s 4-week data collection period, eliminating the potential for alcohol-related discussions on how to make changes in lifestyle or medication-use. Further, participants may not have encountered situations, such as a party or other festive occasion, in which to change their drinking pattern. Additionally, intervention participants may have reported drinking less because they perceived it to be the socially desirable or expected response.

### Limitations

This study’s findings must be interpreted cautiously. The study was an initial test of the website, and there were no comparable studies on which to base sample size calculations. Thus, we cannot be sure if the number of participants was sufficient to detect a true difference between groups. Further, we did not compare “A Toast to Health in Later Life’s feasibility with alternative approaches or programs designed to educate older people about the benefits and risks of alcohol drinking because, to our knowledge, none was available. In fact, it was the lack of existing age-appropriate education that was the impetus for developing and testing “A Toast to Health in Later Life.” We might have developed our own alternative version of the content (eg, audio or print), but that would have necessitated a systematic test of the feasibility and comparability of this new program (eg, for literacy level and age relevance of content), and we did not have the resources to conduct such a study. Instead we took the approach advocated by some researchers who have stated that even if evaluations find that one intervention is superior to an alternative intervention, they cannot claim that the superior treatment should be the new standard because the intervention has not been shown to be superior to the care commonly given by practitioners [87].

This study’s sample included nonhazardous drinkers who may not have had an incentive to change their drinking patterns, raising questions about the appropriateness of including them in the study. We included these participants because previous research has shown that nonhazardous drinking older adults may become risky drinkers over time. In a study of community-dwelling older drinkers, for example, 20 of 112 (18%) of nonhazardous drinkers who did not receive an intervention became hazardous or harmful consumers over the study's 12-month period [67]. Also, because this study aimed to test the website’s feasibility, we considered all current drinkers to be eligible, and the sensitivity analysis did not change the study’s findings. Given that there is evidence that without intervention, some older drinkers may increase their risks, it seems prudent to educate all older drinkers regardless of their current risk. Future research should consider evaluating the effectiveness of patient education in preventing alcohol-related risks in older people as well as reducing those that already exist. Enthusiasm for the applicability of the study’s findings must also be tempered by the fact that the participants were selected because they were comfortable using the Internet. In the United States, nearly 60% of persons 65 and older use the Internet, with persons 55 years and older using it even more frequently [47,88]. These statistics may not apply to other US samples or nations, thereby limiting the feasibility of “A Toast to Health in Later Life” among many older adults.

### Conclusions

This study was designed to test “A Toast to Health in Later Life’s!” feasibility and determine if education has the potential to change older adults’ drinking behavior. We found that the site is usable and acceptable in a White, healthy, and well-educated sample of older people. We do not know if older adults with other demographic characteristics will react to the website in the same positive way. But in terms of its drinking, the study’s sample was comparable to other older adults [2,67] in its frequency of drinking risks and failure to adhere to NIAAA drinking limits. This finding supports the suggestion that the website’s objectives and content are appropriate for a more general US population.

Despite the study’s limitations in setting, duration, and sample characteristics, it is the first that we know of to provide evidence that older adults are willing and able to use online education to learn about alcohol use and aging. Its findings also suggest that for alcohol education to be most effective, it should be included as a component of a larger effort consisting of screening, education and counseling preferably in a health care setting. In the United States, Medicare reimburses health providers who screen older patients and counsel those who are at-risk but do not meet the criteria for alcohol use disorders. Given how many preventive activities are expected of primary care providers [89], online education programs, such as “A Toast to Health in Later Life!” can be used to supplement counseling, possibly ameliorating the health care provider’s burdens especially with respect to sensitive topics such as alcohol use. Important next steps consist of testing the usefulness of “A Toast to Health in Later Life!” or other Web-based alcohol education programs in larger studies on relatively diverse older populations and also evaluating their effectiveness and cost-effectiveness.

## Appendix

Multimedia Appendix 1 A portion of the tables and specifications for the Alcohol-Related Problems Survey.

Multimedia Appendix 2 Informed consent.

## Abbreviations

ARPS: Alcohol-Related Problem Survey
DHSS: Department of Health and Human Services
NIAAA: National Institute of Alcohol Abuse and Alcoholism
NSAID: Nonsteroidal anti-inflammatory drug
