# Supplementary material for: Assessing the Usability of Web-Based Alcohol Education for Older Adults: A Feasibility Study
Source: JMIR Res Protoc. 2016 Feb 1;5(1):e11. doi: 10.2196/resprot.4545 (PMC4754533; doi:10.2196/resprot.4545)
Supplement: Multimedia Appendix 2 [file resprot_v5i1e11_app2.pdf]

Multimedia Appendix 2: Consent to Participate in a Research Project

Dear Participant,

WISE & Healthy Aging is taking part in a research project sponsored by the National Institutes of Health. We would like you to join the project. You have been asked to participate because you 1)are 55 years of age or older; 2) have had one or more drinks containing alcohol in the past 3 months; 3) have an email account and are willing to share your email address with the study team; 4) are comfortable using the Internet 5) have access to high-speed Internet; and 6) are willing to spend about 15 to 30 minutes on two separate occasions one month apart to complete an online alcohol education program and answer online questions in English about alcohol and health.

Your participation in the project is completely voluntary. If you decide not to participate, or if you agree and then change your mind before finishing, the care you receive at WISE will not be affected in any way.

WHAT IS THE PROJECT ABOUT?

As people age, they become more sensitive to alcohol. Alcohol also interacts with medications used by older persons and may affect health and wellbeing. The aim of the project is to see if online education is useful and can increase your understanding of alcohol's influence on health, medication use, and physical and mental functioning among people 55 years of age and older.

WHAT AM I BEING ASKED TO DO?

Half of you will be asked to go online to review an online alcohol education program and then to answer questions also online about your experience and your lifestyle and alcohol use. The other half will be asked to answer questions online about lifestyle and alcohol use.

If you are selected to review the online program, you will be given a password-protected link to “A Toast to Health in Later Life!” and the questions about lifestyle and alcohol. The other people will receive a link to the questions.

We will assign you to your group at random. We will ask all participants to complete questions two times: first, at the beginning of the project and second, about four weeks later.

ARE THERE ANY RISKS OR DISCOMFORTS OF PARTICIPATING IN THIS PROJECT?

The risks of participating in the project include the time you will spend reviewing the alcohol education website or completing the questionnaires. There are no medical procedures involved, and there is no financial cost to you associated with this study.

WHAT ARE THE POSSIBLE BENEFITS?

Benefits of the study are the chance to learn about the possible risks and benefits of alcohol use and the possibility of improving the health education of other older adults. If you choose, we will provide access to the new website to all participants at the conclusion of the study. However, you might not benefit from participation in the study.

WILL I BE PAID FOR MY PARTICIPATION IN THE STUDY?

To partially compensate you for your time, we will pay you $35 after you have completed all online survey questions.

HOW WILL MY PRIVACY BE PROTECTED?

All the information you report is highly confidential and will be used only for study purposes. There are really good methods for protecting your information. We will use all of the following as needed: We will strip off the Internet Service Provider (IP) addresses for data submitted via email or online; the data will be encrypted; a firewall will be used to protect the research computer from unauthorized access; and controlled access privileges will be used on the hardware storing the data. All data you provide will be completely deidentified at the conclusion of the project.

All reports of information gained from your answers to the study's questionnaires will be shown as summaries of all participants' answers. No individually identifying information will ever be used by the project team.

All members of the research team have taken special courses and are certified in protecting your privacy.

When the research is completed, we may save the data for use in future research done by myself or others. We will retain these records for up to 12 months after the study is over. The same measures described above will be taken to protect confidentiality.

WHOM DO I CONTACT IF I HAVE ANY FURTHER QUESTIONS?

You may call Dr. John Beck the Principal Investigator, at 3104XX- 59XX, or email him at egebjcb@ucla.edu Dr. Beck is a geriatrician who specializes in the medical care of older adults and an Emeritus Professor of Medicine at UCLA.

WHAT ARE MY RIGHTS AS A RESEARCH SUBJECT?

You may withdraw your consent at any time and discontinue participation without penalty. You are not waiving any legal claims, rights or remedies because of your participation in this research study.

WHAT IF I AGREE TO BE IN THE STUDY AND THEN CHANGE MY MIND?

You can leave the project at any time. Just close your browser. Again, the care you receive at WISE & Healthy Aging will not be affected in any way if you decide to withdraw from the study.

HOW DO I JOIN THE PROJECT?

If you wish to join the project, please continue to the next page by clicking the SUBMIT button.

THANK YOU VERY MUCH FOR YOUR INTEREST IN THIS PROJECT!
